# Supplementary material for: Impact of Cigarette Smoking on the Expression of Oxidative Stress-Related Genes in Cumulus Cells Retrieved from Healthy Women Undergoing IVF
Source: Int J Mol Sci. 2021 Dec 5;22(23):13147. doi: 10.3390/ijms222313147 (PMC8658611; doi:10.3390/ijms222313147)
Supplement: Supplementary file 1 [file ijms-22-13147-s001.zip › ijms-1462978-supplementary.pdf]

Supplementary Materials

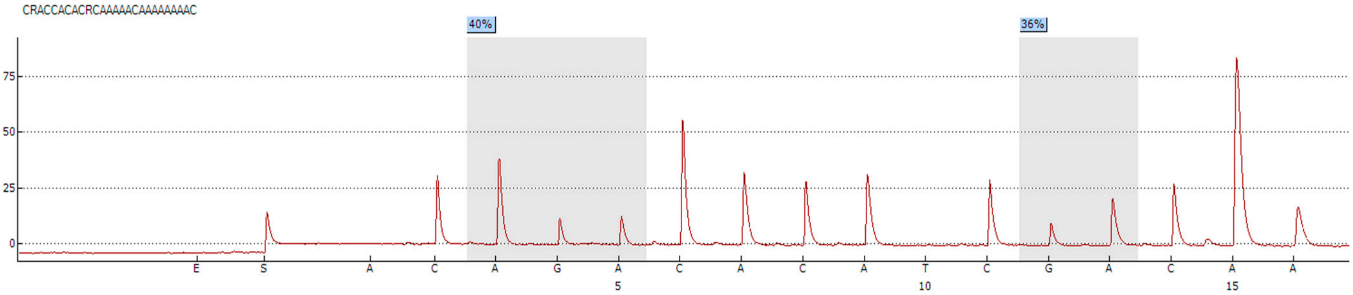

Figure S1. Example pyrogram for IL-6 promoter assay of one of the smoking study participants with mean methylation % approximately equivalent to 38%.

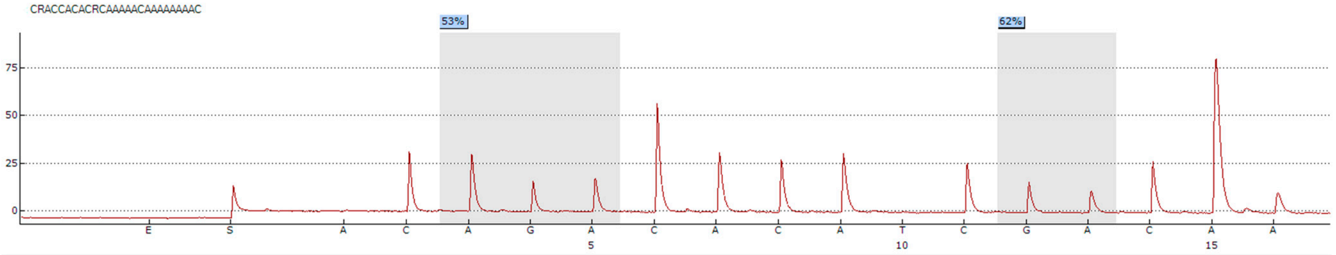

Figure S2. Example pyrogram for IL-6 promoter assay of one of the non-smoking study controls with mean methylation % approximately equivalent to 58%.

Table S1. DNA methylation Primer Sequences and PCR cycling conditions.

**Primer/Sequence to Analyze**	**IL6**
Forward PCR Primer	5'-[Biotin]-TATTTTAGTTTTGAGAA AGGAGGTG-3′
Reverse PCR Primer	5′- CAATACTCTAAAACCCAACAA AAAC - 3′
Sequencing Primer	5′- TCCTAATACAAACAACCCC - 3′
Sequence to Analyze	CRACCACACRCAAAAACAAAAAAAAC
Annealing Temperature (°C)	56 °C for 30 s
